# Expression of fatty acid sensing G-protein coupled receptors in peripartal Holstein cows

**DOI:** 10.1186/s40104-017-0150-z

**Published:** 2017-03-01

**Authors:** Alea Agrawal, Abdulrahman Alharthi, Mario Vailati-Riboni, Zheng Zhou, Juan J. Loor

**Affiliations:** Mammalian NutriPhysioGenomics, Department of Animal Sciences and Division of Nutritional Sciences, University of Illinois, 1207 West Gregory Drive, Urbana, IL 61801 USA

**Keywords:** Inflammation, Methionine, Neutrophils, Transition cow

## Abstract

**Background:**

G-protein coupled receptors (GPCR), also referred as Free Fatty Acid Receptors (FFAR), are widely studied within human medicine as drug targets for metabolic disorders. To combat metabolic disorders prevalent in dairy cows during the transition period, which co-occur with negative energy balance and changes to lipid and glucose metabolism, it may be helpful to identify locations and roles of FFAR and other members of the GPCR family in bovine tissues.

**Results:**

Quantitative RT-PCR (qPCR) of subcutaneous adipose, liver, and PMNL samples during the transition period (-10, +7, and +20 or +30 d) were used for expression profiling of medium- (MCFA) and long-chain fatty acid (LCFA) receptors *GPR120* and *GPR40*, MCFA receptor *GPR84*, and niacin receptor *HCAR2/3*. Adipose samples were obtained from cows with either high (HI; BCS ≥ 3.75) or low (LO; BCS ≤ 3.25) body condition score (BCS) to examine whether FFAR expression is correlated with this indicator of health and body reserves. Supplementation of rumen-protected methionine (MET), which may improve immune function and production postpartum, was also compared with unsupplemented control (CON) cows for liver and blood polymorphonuclear leukocytes (PMNL) samples. In adipose tissue, *GPR84* and *GPR120* were differentially expressed over time, while *GPR40* was not expressed; in PMNL, *GPR40* was differentially expressed over time and between MET vs. CON, *GPR84* expression differed only between dietary groups, and *GPR120* was not expressed; in liver, GPCR were either not expressed or barely detectable.

**Conclusions:**

The data indicate that there is likely not a direct role in liver for the selected GPCR during the transition period, but they do play variable roles in adipose and PMN. In future, these receptors may prove useful targets and/or markers for peripartal metabolism and immunity.

**Electronic supplementary material:**

The online version of this article (doi:10.1186/s40104-017-0150-z) contains supplementary material, which is available to authorized users.

## Background

The G-protein coupled receptor (GPCR) superfamily is one of the largest families of receptor proteins, comprising 1% or more of the human genome, and as much as 5% in simpler organisms like the nematode [1, 2]. GPCR are also termed as seven transmembrane receptors, from their identifying structure of seven α-helices spanning the membrane. They can receive a variety of ligand classes from the extracellular environment, and stimulate intracellular signaling cascades that may begin with action of associated G-proteins [3]. Although GPCR in general are extensively-researched drug targets due to their abundance and activities, those which have metabolic roles may be targets for treating or preventing metabolic and inflammatory diseases in dairy cows, as well. This could especially be of value in the transition period, where prevalence rates and outcomes of diseases are often at their worst [4].

GPCR with fatty acids (FA), and especially longer-chain FA, ligands are among some of the most interesting targets, due to the ability of saturated versus unsaturated fats to evoke different signals within cells [5, 6], and for signaling potency to vary based on chain length and degree of saturation [7–9]. There is also potential to link nutrition, FA metabolism, and immunity among such signaling pathways [10]. For instance, *GPR40* and *GPR120* may be implicated in anti-inflammation in macrophages, but are currently of greater interest for their metabolic effects. These receptors have been connected with obesity, insulin responses, and inflammation subsequent to these conditions [10]. *GPR40* may have a unique contribution to immune function, as it has been shown to stimulate calcium mobilization in bovine neutrophils, a necessary signal for neutrophil activation and function [11]. *GPR84* has been identified in cells of both the innate and adaptive immune system, including PMNL, and plays a role in pro-inflammatory responses, e.g., cytokine production [10]. *GPR109A* also links metabolism and immunity; it has been detected in adipose and macrophages and primarily exerts anti-lipolytic effects [12]. For a summary of these genes and their functions, see Table 1 [10, 11].

**Table 1:** Genes of interest and their functions

Gene Name	Gene Function	References
*GPR40*	M/LCFA receptor expressed in pancreatic α- and β-cells, enteroendocrine cells, immune cells, taste buds, and the central nervous system. Stimulation invokes intracellular calcium response and ERK signaling, or increases in cAMP. Mediates insulin release from β-cells, glucagon release from α-cells, and incretins in the gastrointestinal tract in response to free fatty acid (FFA) ligands, playing a central role in glucose homeostasis. Promotes activation and superoxide production in bovine neutrophils. May regulate secretion of brain-derived neurotrophic factor in neuroblastoma cells.	[8, 11, 38, 48, 66]
*GPR120*	M/LCFA receptor expressed in adipocytes and adipose tissue, macrophages, enteroendocrine cells, pancreatic α-cells, taste buds, and lungs. Stimulation invokes intracellular calcium response and ERK signaling. Stimulation with ω-3 FA leads to β-arrestin2-mediated inhibition of TAK1 (i.e.: anti-inflammatory signaling). Promotes glucose uptake via GLUT4 and G-protein-related insulin stimulatory effects in adipocytes. Promotes incretin (e.g., GLP-1) release in gastrointestinal tract, and glucagon release in α-cells. Reduces inflammatory gene expression in macrophages and adipose, as well as macrophage invasion into adipose tissue.	[38, 44, 49, 67, 68]
*GPR84*	MCFA receptor expressed in adipocytes and immune cells, including leukocytes. Expression is upregulated in macrophages by LPS. Promotes pro-inflammatory cytokine and chemokine signals.	[38, 69, 70]
*HCAR2/3*	Nicotinic acid and butyrate/β-hydroxybutyrate receptor expressed in adipose tissue, immune cells, spleen, colon, pancreatic β-cells, and mammary epithelium. Expression is upregulated in adipose and macrophages by LPS and other pro-inflammatory factors. Activation by niacin or BHBA decreases cAMP levels in adipose tissue, thereby reducing lipolysis, plasma FFA, and availability for triglyceride synthesis. Decreases in cAMP also occur in β-cells, inhibiting insulin release. Promotes release of prostaglandins from immune cells, including macrophages. Invokes apoptotic pathways in neutrophils and some cancer cells (e.g., breast and colon cancer).	[12, 46, 71–73]

The present study also involved an examination of GPCR in adipose tissue, and more specifically cows with different degrees of adiposity as evaluated through the body condition score (BCS) at 21 d prior to parturition. BCS represents energy storage status [13], which is vitally important to the peripartal dairy cow, as early lactation proceeds at the expense of stored energy [14]. What is considered as an optimal score can vary, and may instead be expressed as a range, but is currently thought to be around 3.25 near calving [15]. Generally, however, relatively higher prepartum or calving BCS is correlated with greater BCS [16] and/or body weight [17] loss postcalving. Thus, although all cows experience negative energy balance (NEB) after calving [4], higher BCS cows are at higher risk for deeper NEB and excess lipid mobilization – adverse conditions for an optimal transition. Lower BCS may be considered relatively more optimal for health, although very thin cows have been found to produce less milk [18]. Thus, comparison of FA-sensing receptor expression in both an optimal/thin and a suboptimal/fat group of cows (here, divided as LO = BCS ≤ 3.25, and HI = BCS ≥ 3.75, respectively) could be of value.

The effect of supplementing rumen-protected methionine (MET) was also considered for liver and blood polymorphonuclear leukocytes (PMNL) analyses of GPCR. Previous work by our group demonstrated that MET can improve liver function, immune and antioxidant status, milk yield and protein levels, and may also have beneficial effects on dry matter intake (DMI) around calving [19–21]. Since the GPCR studied here are closely linked to lipid and glucose metabolism and inflammation, it seemed worthwhile to investigate whether gene expression in liver or PMNL is changed with supplementation, and how that relates to observed cow-level effects.

Because the FFAR are not yet well-studied in ruminants [22], the aim of the present study was to assess patterns and levels of receptor expression in periparturient dairy cows. Furthermore, because BCS and supplemental MET can affect production, immune status, and metabolism, we hypothesized that there would be some differential expression between groups.

## Methods

### Animals and treatments

Cows in the present study were a subset from the experiment of Zhou et al. [21]. Cows were blocked according to lactation and calving measures, and fed the same close-up (1.52 Mcal/kg DM; -21 d until calving) and lactation (1.71 Mcal/kg DM; calving until +30 d) diets as a total mixed ration (TMR) once daily (0630 h). Cows were housed in an enclosed, ventilated barn during the dry period and fed using an individual gate system (American Calan Inc., Northwood, NH), and moved to a tie-stall barn with individual feed bunks after calving. Throughout these periods, diets were top-dressed with either no supplement (CON), or Smartamine M (Adisseo NA) rumen-protected methionine (MET). Complete details of supplementation may be found elsewhere [21]. Lactating cows were milked three times daily (0600, 1400, and 2200 h).

Ten and eleven cows from each group (CON and MET) were used in the present study to compare PMNL and liver gene expression, respectively. Additionally, twenty cows that had received adipose biopsies were retroactively grouped by their BCS (see below) at -3 wk from calving (i.e.: upon entry into the close-up dry period), such that 10 cows with BCS ≥ 3.75 (HI; avg. = 3.83 ± 0.12) were compared to 10 cows with BCS ≤ 3.25 (LO; avg. = 3.11 ± 0.16) for adipose gene expression. The effect of supplementation was not considered in this tissue.

### Sample collection

The BCS were assigned weekly during the experiment, along with body weight measurements. BCS was determined on a scale of 1–5 with quarter-point increments, where 1 = thin and 5 = obese. Two scores were given independently each week, so that the average was taken and used for statistical analyses and retroactive grouping. Individual and average BCS loss of cows in each group were also calculated between Prepartum (-21 d) and Postpartum (+21 d) time points. At -10, +7, and +20 d from calving, energy balance (Table 2) was calculated as described previously [23] in the subset of cows which received adipose biopsies.

**Table 2:** Least squares means of energy balance (EB) and energy balance as a percentage of requirements (EB % Req) in transition cows with high (HI; BCS ≥ 3.75) or low (LO; BCS ≤ 3.25) body condition score prepartum and postpartum. This data only represents the subset of cows used for adipose gene expression

	BCS^1^		SEM^2^	*P*-value		
	HI	LO		BCS	Time	BCS × Time
*Prepartum*						
EB	1.89	2.16	1.08	0.86	-	-
EB % Req	112.35	115.62	7.13	0.75	-	-
*Postpartum*						
EB	−11.87^a^	−5.52^b^	2.00	0.03	0.03	0.14
EB % Req	70.85^a^	86.20^b^	4.74	0.03	<0.01	0.30

^a,b^Statistical difference (*P* < 0.05) among time points within the same group

^1^Body condition score

^2^Largest standard error of the mean

Blood was only collected from those cows receiving liver biopsies or scheduled for PMNL retrieval. Serum and plasma were collected for analyses into vacutainers (BD Vacutainer; BD and Co., Franklin Lakes, NJ) containing clot activator or lithium heparin, respectively, at -10, +8, and +30 d relative to parturition. Blood for serum samples was kept at 21 °C, and blood for plasma kept on ice, until centrifugation. Methods for analyses of IL-1β, reactive oxygen metabolites (ROM), and myeloperoxidase (MPO) as indicators of systemic inflammation and oxidative stress was reported previously [20].

Full protocols for relevant tissue biopsies have been previously described [24, 25]. Briefly, cows were given local anesthesia prior to biopsy. The same cows were not used for liver biopsies as for adipose biopsies. Liver was sampled via puncture biopsy at -10, +7, and +30 d from parturition via puncture biopsy, while adipose biopsies from alternate sides of the tail-head region were taken at -10, +7, and +20 d using a blunt dissection method. Both liver and adipose samples were snap-frozen in liquid nitrogen and transferred to a freezer at -80 °C until RNA extraction and further analyses.

### PMN Isolation

Blood samples for collection of PMNL were drawn into vacutainers containing acid citrate dextrose (ACD Solution A; Fisher Scientific) from the coccygeal vein at -10, +7, and +30 d relative to parturition. Samples were placed on ice until PMNL isolation by sample centrifugation, cell lysing, and several rounds of centrifugation with PBS washing. Both purity and viability of PMNL were greater than 90%. Complete details of this process can be found in Zhou et al. [26]. Briefly, a 50 μL aliquot of PMNL was incubated for 15 min on ice with 100 μL of primary anti-bovine granulocyte monoclonal antibody (Cat. No. BOV2067, Washington State University, Pullman, USA) solution (15 μg/mL in 1 × PBS). The aliquot was then washed twice with 2 mL 1 × PBS and incubated for another 15 min on ice, protected from light, with 50 μL of secondary phycoerythrin-labeled secondary antibody (Cat. No. 1020–09S, Southern Biotech, Birmingham, AL) (4 μg/mL in 1 × PBS), and 50 μL of Propidium iodide (50 μg/mL) prior to flow cytometry. Isolated PMNL were homogenized at full speed in a solution of 2 mL TRIzol reagent (Invitrogen, Carlsbad, CA) with 1 μL linear acrylamide (Ambion, Inc., Austin, TX). Homogenate was stored at -80 °C in RNA-free microcentrifuge tubes (Fisher Scientific, Pittsburgh, PA).

### RNA Isolation

To proceed with RNA extraction, 40 mg liver and 200 mg adipose were thawed and homogenized in QIAzol reagent (Qiagen, Hilden, Germany). Extraction of RNA was performed with the miRNeasy kit (Qiagen) following the manufacturer’s protocols. Samples were treated on-column with DNaseI (Qiagen). Prior to storage, RNA purity was confirmed using a NanoDrop ND-1000 (NanoDrop Technologies, Rockland, DE) OD_260nm_/OD_280nm_ ratio, and RNA quality was recorded using a 2100 Bioanalyzer (Agilent Technologies, Inc., Santa Clara, CA) RNA integrity number (RIN). All liver samples had RIN scores above 8.0. Average RIN scores for the other two tissues were as follows: 6.44 ± 0.21 for adipose and 6.68 ± 0.02 for PMNL.

### Real-time Quantitative PCR

Previous publications by our group [27] outlined the full protocols. Briefly, 100 ng of RNA, plus reagents including: 1 μg of dT18 (Operon Biotechnologies, Huntsville, AL), 1 μL of 10 mmol/L dNTP mix (Invitrogen Corp., Carlsbad, CA), 1 μL of random primers (3 mg/μL; Invitrogen Corp.), and 10 μL of DNase-/RNase-free water, were incubated at 65 °C for 5 min, then placed on ice for 3 min. Six μL of master mix, including: 5.5 μL of 5× reaction buffer, 0.25 μL (50 U) of RevertAid reverse transcriptase (Fermentas Inc., Glen Burnie, MD), and 0.25 μL of RNase inhibitor (10 U, Promega, Madison, WI), was then added to complete cDNA synthesis.

Primer design protocols have also been published previously [28]. Except for bovine *GPR40* [29], primer sequences were obtained using Primer Express 3.0. Primer information and obtained products are listed in Additional file 1: Table S1 and S2, respectively. Quantitative PCR was performed using 4 μL of diluted cDNA plus a mixture of 5 μL of 1× SYBR Green master mix (Applied Biosystems, CA), 0.4 μL each of the 10 μmol/L forward and reverse primers, and 0.2 μL of DNase-/RNase-free water in a MicroAmp Optical 384-Well Reaction Plate (Applied Biosystems, Foster City, CA). Each sample was run in duplicate, while a negative control and serially diluted, pooled cDNA were run in triplicate to create a 6-point relative standard curve (User Bulletin #2, Applied Biosystems). PCR reactions were performed in an ABI Prism 7900 HT SDS instrument (Applied Biosystems) under the following conditions: 2 min at 50 °C, 10 min at 95 °C, 40 cycles of 15 s at 95 °C (denaturation), and 1 min at 60 °C (annealing plus extension). Gene expression was normalized using the geometrical mean of three appropriate internal control genes: *GAPDH* and *RPS9* for all tissues, along with *ACTB* for adipose and *UXT* for liver and PMNL [25, 27, 30]. Genes were considered not expressed when the standard curve had slope -3.50 > y > -3.00 and Ct > 30. At least for the Ct threshold, the criterion is consistent with our established protocols aimed in part at reducing the unreliability of data that often occurs at >30 Ct [25, 27, 28, 30]. In that context, it is important to highlight that in recent studies dealing with the study of FFAR the mean Ct for quantification of GPR40 (which was undetectable using our thresholds) ranged from 31.4 for adipose and liver [31] to 35.7 for adipose [32]. Although it could be possible that the size of the amplicon for the GPR40 primer [29] was too long and led to poor amplification efficiency, Yonezawa et al. [29] verified the identity of bovine GPR40 by sequencing (as did we; see Additional Table S2) and used it successfully with bovine mammary tissue. Unfortunately, there was no information on amplicon size for the GPR40 primer used in the work of Friedrichs et al. [31, 32]. The qPCR performance is reported for adipose, liver, and PMNL in Additional file 1: Table S3.

### Statistical analysis

Prior to analysis, expression data were log_2_ normalized. All data sets (i.e.: blood parameters, energy balance, and qPCR) were then subject to ANOVA using repeated measures ANOVA with PROC MIXED in SAS (v 9.2; SAS Institute Inc., Cary, NC). The statistical model for adipose included time (-10, +7, +20 d), BCS (HI, LO), and their interaction as fixed effects. Energy balance was analyzed pre- and postpartum. The model for liver and PMNL gene expression, and blood parameters, included time (-10, +7, +30 d), methionine supplementation (MET, CON), and their interaction as fixed effects. The random effect was cow, nested within treatment. The Kenward-Roger statement was used for computing the denominator degrees of freedom, with sp(pow) as the covariance structure. Energy balance used Compound Symmetry as the covariance structure postpartum. Previous 305 d lactation and parity were used as covariates for blood analysis, and parity was used as a covariate for energy balance. When not significant, covariates were removed from the model. Data were considered significant at *P* ≤ 0.05 using the PDIFF statement in SAS. Expression data in Tables 3 and 5 are reported as the log_2_ back-transformed least squares means.

**Table 3:** Log_2_-backtransformed least squares means of adipose gene expression data in transition cows with high (HI; BCS ≥ 3.75) or low (LO; BCS ≤ 3.25) body condition score at -10, +7, and +20 d from calving

						BCS × Time									
Gene	BCS^1^		Time			HI			LO			SEM^2^	*P*-value		
	HI	LO	−10	+7	+20	−10	+7	+20	−10	+7	+20		BCS	Time	BCS × Time
*GPR40*	Not expressed														
*GPR84*	0.37	0.41	0.19^a^	0.69^b^	0.46^b^	0.21^a^	0.39^a,b^*	0.61^b^	0.17^a^	1.21^b^*	0.34^a^	0.40	0.62	<0.01	0.03
*GPR120*	0.46	0.72	1.69^a^	0.49^b^	0.22^c^	1.36	0.37	0.19	2.09	0.67	0.26	0.64	0.08	<0.01	0.89
*GPR109A*	1.05	1.30	1.12	1.40	1.02	1.13	1.16	0.89	1.10	1.68	1.17	0.22	0.06	0.08	0.33

^a,b,c^Statistical difference (*P* < 0.05) among time points within the same group

*Statistical difference (*P* < 0.05) between groups within time points

^1^Body condition score

^2^Largest standard error of the mean

## Results

### Body condition score experiment

#### Energy balance

Although there was no difference in energy balance by BCS prepartum (*P* = 0.86) (Table 2), cows in the HI BCS group were in more NEB (*P* = 0.03) during the postpartum period.

### Adipose

*GPR40* was not expressed in adipose tissue. *GPR84* expression was significantly lower (*P* < 0.05) prepartum than postpartum (Table 3). There was also an interaction effect (*P* = 0.03) of BCS × time, such that expression was higher in LO than in HI cows at +7 d. There was also a significant time effect (*P* < 0.01) on expression of *GPR120*, where expression was highest prepartum, and decreased at both of the postcalving time points. Expression of both *GPR120* and *GPR109A* tended (*P* = 0.08 and 0.06) to be greater in LO than in HI. There was also a tendency (*P* = 0.08) due to the time effect on *GPR109A*, mainly for the difference between +7 and +20 d.

### Rumen-protected methionine experiment

#### Inflammation and oxidative stress biomarkers

Overall, cows in MET had lower (*P* < 0.05) concentrations of IL-1β and ROM but had greater (*P* < 0.05) concentration of MPO (Table 4).

**Table 4:** Least squares means of immune biomarker concentrations in blood in transition cows supplemented with rumen-protected methionine (MET) or unsupplemented (CON) at -10, +7, and +30 d. This data only represents the subset of cows used for polymorphonuclear leukocyte (PMNL) gene expression

Parameter	Diet		SEM^1^	*P*-value		
	MET	CON		Diet	Time	Diet × Time
IL-1β^2^	3.63^a^	5.74^b^	0.69	0.05	0.17	0.70
ROM	12.29^a^	14.20^b^	0.23	<0.01	<0.01	0.05
MPO	466.87^a^	405.18^b^	15.89	0.02	0.14	0.44

^a,b^Statistical difference (P ≤ 0.05) among time points within the same group

^1^Largest standard error of the mean

^2^Significance (*P* < 0.05) for parity in the model

### Liver

Neither *GPR40* nor *GPR120* were expressed in liver (Table 5). For *GPR84* and *GPR109A*, there were no significant differences (*P* > 0.15) or tendencies for effects of MET, time, or the interaction.

**Table 5:** Log_2_-backtransformed least squares means of liver and polymorphonuclear leukocyte (PMNL) gene expression data in transition cows supplemented with rumen-protected methionine (MET) or unsupplemented (CON) at -10, +7, and +30 d from calving

						Diet × Time									
Gene	Diet		Time			MET			CON			SEM^1^	*P*-value		
	MET	CON	−10	+7	+30	−10	+7	+30	−10	+7	+30		Diet	Time	Diet × Time
Liver															
*GPR40*	Not expressed														
*GPR84*	0.77	0.84	0.88	0.74	0.80	0.85	0.73	0.75	0.91	0.75	0.86	0.14	0.64	0.25	0.89
*GPR120*	Not expressed														
*GPR109A*	0.51	0.53	0.64	0.48	0.46	0.57	0.52	0.44	0.72	0.44	0.47	0.15	0.77	0.22	0.61
PMNL															
*GPR40*	0.85*	1.13*	1.15^a^	0.95^a,b^	0.85^b^	1.00	0.80	0.77	1.33	1.14	0.94	0.17	0.04	0.04	0.78
*GPR84*	1.49*	0.65*	0.96	1.05	0.96	1.22	1.82	1.49	0.75	0.60	0.61	0.64	0.04	0.93	0.51
*GPR120*	Not expressed														
*GPR109A*	1.34	0.87	1.26	0.91	1.10	1.42	1.31	1.28	1.11	0.63	0.94	0.44	0.09	0.60	0.71

^a,b^Statistical difference (*P* < 0.05) among time points within the same group

*Statistical difference (*P* < 0.05) between groups within time points

^1^Largest standard error of the mean

### PMNL

*GPR120* was not expressed in PMNL (Table 5). Among the other three genes, there were significant differences (*P* < 0.05) between MET and CON for *GPR40* and *GPR84*, such that *GPR40* was lower in MET, and *GPR84* was higher in MET. *GPR40* expression also differed across time (*P* = 0.04), such that cows had significantly greater expression at -10 d than at +30 d, decreasing (albeit not significantly) in between. *GPR109A* tended (*P* = 0.09) to be higher in MET PMNL than in CON.

## Discussion

### Body condition score

#### Adipose tissue

Postpartum, transition cows can experience stress-induced, pathogen-independent inflammation, as marked by pro-inflammatory cytokines and acute-phase proteins (APP) in the blood [33]. Furthermore, expression of chemoattractants and cytokines within adipose tissue indicates some degree of local inflammation, which has been postulated as a homeorhetic mechanism to aid lactation [34, 35]. This theory is supported by the knowledge that cytokines present during inflammation encourage lipolysis [34], the “metabolic hallmark” of transition [36]. Here, both the function and the temporal expression of *GPR84* match physiologic adaptations in the cow, indicating that effects of inflammation in adipose are partly mediated through this receptor.

An interaction of BCS × time at +7 d, where LO cows had much greater expression of *GPR84* than HI cows also provides evidence of the pro-inflammatory role for GPR84 in cattle. Biomarkers like haptoglobin, bilirubin, and paraoxonase have been used to indicate peripartal inflammation through the first 2-3 wk of lactation [37]. Here, although blood biomarker data were unavailable, the surge in *GPR84* expression may also indicate greater inflammation in LO cows, at least within the adipose tissue depot [38]. This may not necessarily be mirrored at a systemic level since cows with higher BCS are normally associated with greater overall inflammation and lower health status [13]. Despite this, thin cows can still be health-compromised during the transition period [13, 39]. Barring clinical or subclinical disease, inflammatory signals resolve toward the end of the transition period [37], which parallels the return of *GPR84* expression to prepartum levels.

If the above holds true, GPR84-mediated inflammation could also help explain production in these cows. Inflammation in early lactation has been connected with poorer performance (i.e. milk yield; [33]), and in fact, through the first month, milk yield from LO cows was numerically, albeit not statistically, lower than HI cows (39 kg/d vs. 42 kg/d; data not shown). In a recent study [17], Pires et al. obtained similar results: numerically, low BCS cows had lower production than medium or high BCS cows. Further work including more cows, as well as immune biomarkers concurrent with gene expression, will be necessary to elucidate the potential involvement of *GPR84* and validity of such relationships.

Contrary to GPR84, GPR120 is primarily an anti-inflammatory receptor [8]. Thus, lower expression over time potentially reinforces evidence of postpartal inflammation, although without a lessening effect by 3 week postpartum. Its metabolic role may explain this pattern: GPR120 stimulates adipogenesis and differentiation, rather than lipolysis [40]. As necessity for lipolysis increases through early lactation NEB [14], expression of *GPR120* should decrease, as observed here. Receptor activation in adipose also improves insulin sensitivity [41]. Because insulin resistance in peripheral tissues is important for pushing available glucose to the mammary gland post-calving [42], lower expression of *GPR120* in adipose should be expected, and may be a necessary part of the transcriptome adaptation to milk synthesis.

To a lesser extent, limited expression could also be a regulatory mechanism. As lipolysis occurs, releasing primarily long-chain FA (LCFA) (i.e. GPR120 ligands) to the blood [43], signaling may provide negative feedback to prevent hyperactivation. In some cases, GPR120 can activate β-arrestin2, which promotes receptor internalization and prevents continuous ligand-sensing [44]; perhaps gene expression is used as an additional level of control, or acts as a primary regulatory mechanism when alternate pathways are activated.

Both of the above ideas may explain why HI cows tended to have lower overall *GPR120* expression. As previously mentioned, milk yield of HI cows was ~3 kg/d greater, numerically. Greater milk production translates to greater glucose requirements [36], and gene expression should reflect a heightened need for peripheral insulin resistance. To produce milk during NEB, HI cows may also mobilize more of their body fat reserves which we also detected in this study, leading to higher FA mobilization and circulating FA [16, 39]. The marked NEB postpartum in HI cows supports this idea. As GPR120 interacts with free LCFA, a negative response on gene expression may prevent further signaling at a time when that could be counterproductive. Certainly, going forward, this receptor could be an interesting metabolic target in the transition period.

The finding that *GPR109A* tends to have greater expression in LO than in HI cows is not surprising. Studies of *GPR109A* indicated that it is a primary anti-lipolytic receptor in adipose tissue [45]. The lower BCS in LO cows was indicative of potentially lower amount of stored fat, and greater expression of this receptor could be a mechanism to maintain as much of their already-reduced body condition as possible. Smaller BCS losses between the beginning of the close-up period (-21 d) and both postpartum time points (+21 d) in LO cows compared with HI cows may provide evidence in support of this hypothesis (BCS loss of 0.80 versus 0.40). To address the species-specific effects of *GPR109A* on lipolysis, that is, whether in ruminants it has temporary and/or rebound effects as in humans, or longer-term FA-lowering effects as in rodents [46], plasma FA from multiple time points should be considered. Although no blood data were available for the subset of cows with adipose biopsies, amount of circulating FA can be correlated with energy balance (EB) [36]. Thus, with better EB (i.e.: more shallow NEB) than HI cows at both time points postpartum, it can be assumed that LO cows had lower circulating FA, meaning that *GPR109A* may be effective over some period of time in cattle. Blood analyses will be useful to confirm this idea.

For this reason, a tendency for greater *GPR109A* expression +7 d after calving, regardless of BCS, was unexpected. The nadir in energy balance, when low DMI coincides with high lactation requirements, typically occurs within three weeks following calving. As previously mentioned, this tends to correspond with lipomobilization; in fact, peak levels of basal and norepinephrine-stimulated lipolysis occur around +10 d [14]. Thus, it appears that high rates of lipolysis can occur in spite of the anti-lipolytic influence of *GPR109A*.

This could be due to a greater net signal for fat mobilization over storage brought on by calving and initiation of lactation. Because *GPR109A* signaling involves a decrease in cAMP levels that inhibits hormone-sensitive lipase and prevents release of FA [45], it is possible that parturition- and lactation-induced flux in hormone levels themselves (e.g., increased catecholamines and decreased insulin) are more influential on hormone-sensitive lipase than GPCR signaling [47]. More information could become available with trials on *GPR109A* in periparturient animals.

### Rumen-protected methionine supplementation

#### Liver

It is not altogether surprising that the genes of interest were not expressed, or were expressed at low levels in the liver. Although these GCPR can be widely expressed throughout the body [8], few studies identified the liver as a major site of expression for these particular genes. In fact, *GPR40* [46, 47] and *GPR109A* [43, 48] have been reported as not detected in liver, and *GPR120* has been reported as not detected, except in Kupffer cells (i.e.: macrophages), in the liver [49, 50]. In the present study, the fact that *GPR40* and *GPR120* were not detected agrees with the literature.

The barely detectable expression of *GPR109A* and *GPR84*, and the lack of differences between groups or across time, indicates that these genes may typically be expressed at very low levels, if at all, in bovine liver. Greater numbers of cows, and/or protein detection methods, should be used in future studies to confirm the presence or absence of these receptors within the liver.

Nonetheless, the genes of interest do play an important role in function and diseases in the liver. It is therefore plausible that effects of these GPCR on the liver are mostly indirect, as a result of signaling that originates in other peripheral tissues or immune cells. Notably, the acute, insulin-promoting effects of GPR120 [41], and GPR109A signaling to reduce hormone-sensitive lipase [45] in adipose tissue, could protect against excess lipid mobilization preceding fatty liver. Conversely, activation of GPR40 in pancreatic β-cells could signal hyperinsulinemia and increased risk of lipid accumulation in the liver [51]. Therefore, for the present genes of interest, systemic metabolic networks – rather than localized pathways – may provide better insight as to hepatic responses in bovines. Alternatively, other families of receptors, e.g., peroxisome proliferator-activated receptor (PPAR), could contribute more to direct outcomes of relevant ligands (i.e.: LCFA) in the liver [52].

#### PMNL

Neutrophils are the only tissue in which *GPR40* was detected. Since GPR40 has already been implicated in calcium-dependent degranulation and superoxide production in bovine neutrophils [53], its presence was certainly expected. Its downregulation over time suggests lower PMNL activity postpartum. Indeed, it is well-known that hormones and NEB contribute to immunosuppression post-calving [54–56]. However, it is curious that MET cows had lower expression than CON despite having greater phagocytosis and oxidative burst capacity when challenged in vitro with a bacterial pathogen [20] and higher plasma levels of myeloperoxidase (Table 4) [20]. Instead, it seems that MET could indirectly affect *GPR40* expression through substrate (i.e. fatty acid) availability, because inflammation can promote lipolysis [57], but MET supplementation appears to benefit the inflammatory status (i.e. lower IL-1 β and ROM) [34]. However, MET supplementation did not affect circulating FA levels [21]. Thus, the role of MET supplementation in regulating *GPR40* expression needs further investigation.

In contrast, *GPR84* upregulation in MET agrees with previous data describing enhanced immune response (i.e.: phagocytosis and respiratory burst) in these cows [20]. *GPR84* expression can be stimulated by lipopolysaccharide (LPS), and subsequent signaling produces pro-inflammatory signals, demonstrating that receptor function is tied to immune responses [10]. In agreement, in vivo data revealed that supplementation with MCFA (i.e.: GPR84 ligands) in the transition period lessened neutrophil apoptosis [58], thereby improving antimicrobial capacity of the cells [59]. S Piepers and S De Vliegher [58] hypothesized that GPCR signaling could be at least partly responsible for the improvement in cell viability, and the present study provides evidence that GPR84 could specifically play a role.

Interestingly, *GPR109A* also tended to be upregulated in MET PMNL vs. CON. Like *GPR84*, *GPR109A* expression can be induced by LPS [12], yet with opposite outcome: *GPR109A* activates apoptosis in neutrophils [60]. As noted above, apoptosis should correspond with lower PMNL function, but this did not occur. On the other hand, because apoptosis promotes resolution of inflammation [61], *GPR109A* expression concurs with the largely anti-inflammatory environmental conditions. Possibly, with the influence of MET to lower inflammation (as demonstrated by Zhou et al. [21]), neutrophils maintain their function over a short lifespan [62]. To aid in rapid clearance of aged neutrophils and maintenance of a stable environment, MET cells would induce apoptosis versus necrosis [61]. In this way, *GPR109A* may help protect transition cows from chronic inflammation. More in-depth studies would be needed, but if true, this could provide a new context in which to study niacin as a transition feed supplement [63–65].

## Conclusions

Conditions which are present in ruminants and cause metabolic disorders and clinical disease near calving: negative energy balance, lipid mobilization, insulin resistance, and immunosuppression, closely resemble dysregulated metabolic systems in human diseases. Thus, molecular targets in human medicine may translate as targets for the transition cow. The present GPCR show promise for such work. Although none were expressed well in the liver, their contributions to inflammation, insulin resistance, and lipolysis in adipose indicate that they may indirectly affect liver accumulation of fat. The GPCR-modulated environment may also contribute to level of milk production and severity of systemic inflammation in early lactation. Additionally, the differences between BCS groups highlight the role of the transcriptome in coordinating lipid metabolism and energy status, and differences between MET and CON reinforce previous findings and demonstrate potential networks for immune-enhancing action of supplemental methionine. Thus, the present GPCR and related receptors (e.g., *FFAR2* and *FFAR3*) are suggested as continued areas of research in bovine to improve transition health.

## Additional file

Additional file 1:
**Table S1.** Forward and reverse primer information for genes of interest. **Table S2.** PCR product sequences obtained using primers listed in Table 2. **Table S3.** qPCR performance of *GPR40*, *GPR120*, *GPR84*, and *HCAR2/3* in adipose, liver, and polymorphonuclear leukocytes (PMNL). (DOCX 16 kb)

## Abbreviations

APP: Acute phase protein
BCS: Body condition score
CON: Control-fed
DMI: Dry matter intake
EB: Energy balance
FA: Fatty acid
FFAR: Free fatty acid receptor
GPCR/GPR: G-protein coupled receptor
HCAR: Hydroxycarboxylic acid receptor
HI: High BCS
IL-1B: Interleukin 1β
LCFA: Long-chain fatty acids
LO: Low BCS
LPS: Lipopolysaccharide
MCFA: Medium-chain fatty acids
MET: Methionine-supplemented
NEB: Negative energy balance
PMNL: Polymorphonuclear leukocytes
PPAR: Peroxisome proliferator-activated receptor
qPCR: quantitative polymerase chain reaction
RIN: RNA integrity number
ROM: Reactive oxygen species
TMR: Total mixed ration.
