# Supplementary material for: Expression of fatty acid sensing G-protein coupled receptors in peripartal Holstein cows
Source: J Anim Sci Biotechnol. 2017 Mar 1;8:20. doi: 10.1186/s40104-017-0150-z (PMC5331663; doi:10.1186/s40104-017-0150-z)
Supplement: Additional file 1: — Table S1. Forward and reverse primer information for genes of interest. Table S2. PCR product sequences obtained using primers listed in Table 2. Table S3. qPCR performance of GPR40, GPR120, GPR84, and HCAR2/3 in adipose, liver, and polymorphonuclear leukocytes (PMNL). (DOCX 16 kb) [file 40104_2017_150_MOESM1_ESM.docx]

**Additional Table S1.** Forward and reverse primer information for genes of interest.

| **Accession Number** | **Gene Name** | **Forward Sequence** | **Reverse Sequence** |
| --- | --- | --- | --- |
| XM_015475510.1 | *HCAR2/3* | ACATCACCCTCAGCTTCACC | GCGGTTGTTATCCGACTCAT |
| NM_001309646.1 | *GPR40* | TCTATGTGGCTGCCTTCGTG | GAGCAGAAGCGAGAGGCTAA |
| NM_001038568.1 | *GPR84* | TTCCGCTGAAGAGAGATTTGAAG | GGTAGCAGGGAGAAGTTGACGTCAGA |
| NM_001328657.1 | *GPR120* | TTGGTACCAGGACTGCTTATTGTG | ATCGTCAGCCTCTTCCTGGAA |

**Additional Table S2.** PCR product sequences obtained using primers listed in Table 2.

| **Primer Name** | **Obtained Sequence** |
| --- | --- |
| *HCAR2/3* | CTTTGGTGTACTACTTCTCCAGCCCATCTTTCCCCAACTTCTTCTCCACCTTGATCAACCGCTGCCTGAAGAGGAAGGGGCCGGATGAGTCGGATAACAACCGCA |
| *GPR40* | CATCGCGGCGCGTGTCCCACGCCCGGCTGCGCCTCACCCCCAGCTTGGTCTATGCCCTCCACCTGGGCTGCTCTGATCATACCTGCTGGCGACTTCTCTGCCCCTGAAGGCAGTGGAGGCCCTGGCTGGGGGCACCTGGCCCCTGCCGGCCCCTCTCTGTCCTGCCTTCGCCCTGGTCCACTTCGCTCCACTCTATGCGGGAGGGGGCTTCCTGGCCGCCCTGAGTGTTGGCCGCTACCTCGGAGCTGCCTTCCCCTTGGGCTACCAAGCTGCCCGGAGGCCGCTCTACTCCTGGGGCGTGTGTGTGGCCGTATGGGCCATCGTTCTCTGTCACCTGGGGCTGGTCTTTGGGCTGGAGGCCCCGGGGGGCTGGCTGGACAATTCCACCAGCTCCTTGGGCATCAGCACACCAATCAATGGCTCTCCGGTCTGCCTGGAGGCCTGGGACCCAGCATCGGCAGGCCCGGCTCGCTTTAGCCTCTCGCTTCTGCTCA |
| *GPR84* | GCATGCTCTCATCATGTGACGCTTCTGACGTCACTTCTCCTGGCATACTAGA |
| *GPR120* | ATCAGTTACTCCAAAATTTTACAGATCACGAAGGCTTCCAGGC |

**Additional Table S3.** qPCR performance of *GPR40*, *GPR120*, *GPR84*, and *HCAR2/3* in adipose, liver, and polymorphonuclear leukocytes (PMNL).

| **Gene** | **Median Ct**^1^ | **Median ∆Ct**^2^ | **Slope**^3^ | **(R^2^)**^4^ | **Efficiency**^5^ | **Relative mRNA Abundance**^6^ | **1/E∆Ct**^7^ | **% mRNA Abundance**^8^ |  |
| --- | --- | --- | --- | --- | --- | --- | --- | --- | --- |
| **Adipose** |  |  |  |  |  |  |  |  |  |
| *GPR40* | 29.86 | 8.82 | -0.18 | 0.40 | 270358.48 | 0.000 | 0.000 | 0.00 |  |
| *GPR84* | 30.57 | 9.61 | -3.08 | 0.98 | 2.11 | 0.001 | 0.023 | 2.34 |  |
| *GPR120* | 28.61 | 7.37 | -3.45 | 0.99 | 1.95 | 0.007 | 0.228 | 22.83 |  |
| *HCAR2/3* | 26.40 | 5.57 | -3.44 | 0.99 | 1.95 | 0.024 | 0.748 | 74.84 |  |
| **Liver** |  |  |  |  |  |  |  |  |  |
| *GPR40* | 29.34 | 7.87 | -0.41 | 0.72 | 268.72 | 0.000 | 0.000 | 0.00 |  |
| *GPR84* | 32.77 | 11.28 | -3.32 | 0.97 | 2.00 | 0.000 | 0.548 | 54.82 |  |
| *GPR120* | 33.07 | 11.75 | -1.57 | 0.97 | 4.32 | 0.000 | 0.000 | 0.00 |  |
| *HCAR2/3* | 31.28 | 9.69 | -2.78 | 0.98 | 2.29 | 0.000 | 0.452 | 45.17 |  |
| **PMNL** |  |  |  |  |  |  |  |  |  |
| *GPR40* | 22.91 | 2.84 | -2.99 | 1.00 | 2.16 | 0.112 | 0.389 | 38.92 |  |
| *GPR84* | 23.76 | 3.90 | -3.05 | 1.00 | 2.13 | 0.053 | 0.183 | 18.32 |  |
| *GPR120* | 31.72 | 12.05 | -0.80 | 0.26 | 17.48 | 0.000 | 0.000 | 0.00 |  |
| *HCAR2/3* | 23.08 | 3.08 | -3.38 | 0.99 | 1.98 | 0.123 | 0.428 | 42.75 |  |
| ^1^ The median is calculated considering all time points and all cows  ^2^ The median of ∆Ct is calculated as [Ct gene – geometrical mean of Ct internal controls] for each time point and each cow  ^3^ Slope of the standard curve  ^4^ R^2^ stands for the coefficient of determination of the standard curve  ^5^ Efficiency is calculated as [10(-1/Slope)]  ^6^ Relative mRNA abundance = 1/Efficiency Median ∆Ct  ^7^ 1/E∆Ct = relative mRNA abundance/∑ relative mRNA abundance  ^8^ Percent mRNA abundance = 100(1/E∆Ct) | | | | | | | | | |
